# Sphingolipid profiling as a biomarker of type 2 diabetes risk: evidence from the MIDUS and PREDIMED studies

**DOI:** 10.1186/s12933-024-02505-7

**Published:** 2024-12-18

**Authors:** Loni Berkowitz, Cristina Razquin, Cristian Salazar, Fiorella Biancardi, Ramón Estruch, Emilio Ros, Montserrat Fitó, Dolores Corella, Christopher L. Coe, Carol D. Ryff, Miguel Ruiz-Canela, Jordi Salas-Salvado, Daniel Wang, Frank B. Hu, Amy Deik, Miguel A. Martínez-Gonzalez, Attilio Rigotti

**Affiliations:** 1 Department of Nutrition, Diabetes and Metabolism, School of Medicine, Pontificia Universidad Católica of Chile, Diagonal Paraguay #362, Santiago, Chile; 2 Department of Preventive Medicine and Public Health, IdiSNA, University of Navarra, Pamplona, Spain; 3 Department of Internal Medicine, Institut d’Investigacions Biomèdiques August Pi Sunyer Biomedical Research Institute (IDIBAPS), Hospital Clinic, University of Barcelona, Barcelona, Spain; 4 Department of Preventive Medicine and Public Health, University of Valencia, Valencia, Spain; 5 Lipid Clinic, Department of Endocrinology and Nutrition, August Pi Sunyer Biomedical Research Institute (IDIBAPS), Hospital Clinic, University of Barcelona, Barcelona, Spain; 6 Spanish Biomedical Research Centre in Physiopathology of Obesity and Nutrition (CIBEROBN), Health Institute Carlos III, Madrid, Spain; 7 Department of Psychology, University of Wisconsin-Madison, Madison, WI USA; 8 Institute on Aging, University of Wisconsin-Madison, Madison, WI USA; 9 Unitat de Nutrició Humana, Departament de Bioquímica i Biotecnologia, Grup d’Alimentació, Desenvolupament i Salut Mental (ANUT-DSM), Universitat Rovira i Virgili, Reus, Spain; 10 Institut d’Investigació Sanitària Pere Virgili (IISPV), Reus, Spain; 11 Department of Nutrition, Harvard T.H. Chan School of Public Health, Boston, MA USA; 12 Channing Division of Network Medicine, Department of Medicine, Brigham and Women’s Hospital and Harvard Medical School, Boston, MA USA; 13 Department of Epidemiology, Harvard T.H. Chan School of Public Health, Boston, MA USA; 14 The Broad Institute of Harvard and MIT, Boston, MA USA

**Keywords:** Type 2 diabetes, Sphingolipids, Ceramides, Lactosylceramides, Insulin resistance

## Abstract

**Background:**

Type 2 diabetes (T2D) has become a worldwide pandemic. While ceramides may serve as intermediary between obesity-related lipotoxicity and T2D, the relationship with simple glycosphingolipids remains uncertain. The aim of this study was to characterize the associations between blood glycosphingolipid and ceramide species with T2D and to identify a circulating sphingolipid profile that could serve as novel biomarker for T2D risk.

**Methods:**

Cross-sectional relationship between sphingolipid levels, insulin resistance, and T2D prevalence were evaluated in 2,072 American adults from MIDUS cohort. Prospectively, the association between sphingolipid species and the incidence of T2D was analyzed using a case-cohort design nested within the PREDIMED trial (250 cases and a random sample of 692 participants, with 3.8 years of median follow-up). Circulating levels of sphingolipid species in both populations were measured using LC/MS. Hazard ratios were estimated with weighted Cox regression models using Barlow weights.

**Results:**

In American adults, only CER18:0 and CER22:0 were linked to insulin resistance and a higher prevalence of T2D. Conversely, three lactosylceramides (LCER 14:0, 16:0, and 24:1) showed a strong inverse relationship with both insulin resistance and T2D. These findings led to development of two sphingolipid scores. In the prospective analysis, these scores consistently predicted a reduced risk of T2D incidence in PREDIMED (HR: 0.64, 95% CI 0.44 to 0.94 and 0.58, 0.40 to 0.85 respectively) between extreme quartiles, with 5-year absolute risk differences of 9.6% (95% CI: 0.3–20.5%) and 11.4% (1.0–21.6%). They were validated in the same trial with samples obtained after 1 year of follow-up.

**Conclusions:**

Our findings support the potential usefulness of circulating sphingolipid profiles as novel biomarkers for T2D risk. Moreover, this study opens the door for future research on the predictive value and possible protective roles of lactosylceramides in T2D.

**Graphical abstract:**

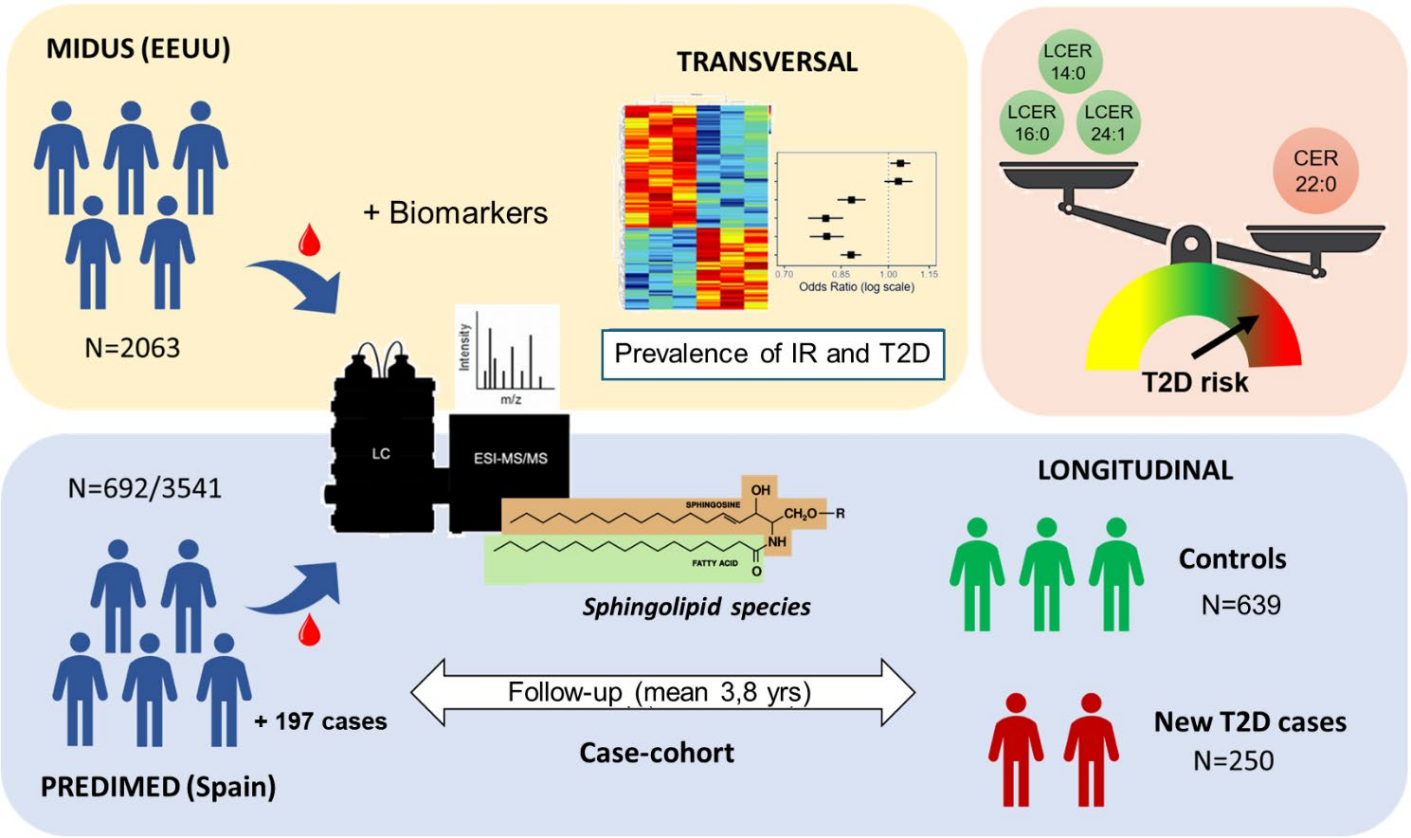

**Supplementary Information:**

The online version contains supplementary material available at 10.1186/s12933-024-02505-7.

## Background

Type 2 diabetes mellitus (T2D) is a metabolic disorder that has become a global pandemic and a major healthcare burden worldwide. T2D has a global prevalence of 9%, and it is projected that there will be > 600 million patients diagnosed by 2040 [1]. T2D is mainly caused by insulin resistance as well as impaired insulin production and secretion. There are multiple risk factors, and a primary one is obesity-related lipotoxicity. The accumulation of fat in ectopic sites results in lipotoxicity, negatively affecting pancreatic beta-cell function and peripheral insulin sensitivity [2].

Among the numerous lipid subtypes that contribute to lipotoxicity, the impact of ceramide accumulation has particularly deleterious effects [3]. Ceramides, which are members of the sphingolipid family and precursors of complex sphingolipids, may have a causal role in diabetes [4, 5]. Studies have shown that ceramides directly affect insulin signaling, leading to insulin resistance [6–9].

In contrast to unglycosylated ceramides, the involvement of lactosylceramides and other simple glycosphingolipids in cardiometabolic diseases is more controversial. While circulating levels of both ceramide and lactosylceramide have been directly associated with inflammatory processes and cardiovascular disease [10–13], some studies have shown an inverse relationship between plasma levels of lactosylceramides and insulin resistance, or with the incidence of diabetes [14, 15]. Even though the inflammatory and oxidative effects of lactosylceramides may exacerbate disease progression in patients who have already developed diabetes (reviewed in [16]), the role of lactosylceramides in the development of new-onset T2D and their prognostic value remain unknown.

The objective of this study was to further characterize the associations between insulin resistance, T2D and sphingolipid profiles in circulation, with an emphasis on ceramide and lactosylceramide species. Following an initial analysis of American adults, the validity of the findings was tested through a prospective examination of these associations to assess new T2D cases in Spanish adults.

## Methodology

### Design

An observational cross-sectional study was conducted using data from the Midlife in the United States (MIDUS) project [17] to characterize the sphingolipid profile associated with insulin resistance and T2D. Subsequently, an unstratified nested case-cohort analysis was conducted within the *Prevención con Dieta Mediterránea* (PREDIMED) trial [18, 19] to assess the longitudinal association between the plasma sphingolipid pattern at baseline with the incidence of new-onset T2D.

### MIDUS: study sample and variables

#### Study sample from MIDUS

Participants were middle-aged and older Americans evaluated during the MIDUS 2 and MIDUS Refresher phases. MIDUS was initiated in 1995/96 to assess the role of behavioral, psychological, and social factors in adult health. MIDUS 2 was a longitudinal follow-up conducted approximately 9–10 years after the baseline survey and included comprehensive biomarker assessments of a subsample [17]. The MIDUS Refresher broadened the age and racial representation by recruiting a new set of participants after the 2007–2009 Great Recession [20].

From subjects who participated in the Biomarker project (n = 2,118; 1,255 from MIDUS 2 and 863 from MIDUS Refresher), we excluded 46 individuals (2.2%) who did not have lipidomic data or metabolic measures. Thus, the current analysis included 2,072 subjects (54.7% females, 74.9% Whites of European family backgrounds, age = 55.6 ± 12.5 years) who participated in the MIDUS 2 (n = 1,220) and MIDUS Refresher (n = 852) biomarker assessments. Additional information about this cohort is provided elsewhere [21].

#### Sociodemographic, anthropometric, and biochemical variables in MIDUS

Sociodemographic variables included age, biological sex, race, and years of education. Race was ascertained by self-identification. Health-related variables included body mass index (BMI, kg/m^2^), waist circumference (cm), low density lipoprotein cholesterol (LDL-c, mg/dL), high density lipoprotein cholesterol (HDL-c, mg/dL), triglycerides (mg/dL), total cholesterol (mg/dL), plasma glucose (mg/dL) and insulin (uIU/mL), hemoglobin A1c (%), and HOMA-IR.

BMI and waist circumference were assessed by trained clinical personnel at one of the three Clinical and Translation Research Centers (University of Wisconsin-Madison, UCLA, and Georgetown University) during participants’ overnight visits. Information about medication usage was acquired during the visit for blood collection.

All biochemical measurements were derived from a fasting blood sample, as described elsewhere [22]. A standard lipid panel (including HDL-c, calculated LDL-c, and triglyceride levels) and measures of gluco-regulation (plasma glucose, insulin, HOMA-IR, and hemoglobin A1c) were analyzed by certified clinical laboratories operating under CLIA standards.

#### Dietary assessment in MIDUS

To assess the influence of saturated fat intake on sphingolipid levels, dietary intake data was used. Food intake data were obtained via medical history interview conducted by project staff during the biomarker clinic visit. Participants were asked how many servings and how often they consumed a specific food group (fast food and high fat meat) during an average week. A score of 0 to 1 was assigned to each category, with 0 indicating no intake, 0.5 for low intake (high-fat meat 1–2 servings/week, fast food ≤ 1 times/week), and 1 point for high intake (high-fat meat > 3 servings/week and fast food > 1 time/week). Typical serving sizes were pre-specified.

#### Sphingolipid profiling in MIDUS

Serum samples were obtained at all 3 biomarker sites using standardized protocols and shipped frozen to Metabolon, Inc. (Durham, NC), where sphingolipid profiling was performed as part of an untargeted-lipidomic analysis. The protocol used for the collection and processing of serum samples, as well as lipid quantification, is explained in detail in Supplementary material. Briefly, LC-MS techniques were used to quantitatively profile lipid species as described elsewhere [23]. Quantitation was achieved using class-specific internal standards, with each lipid class having one or more labeled internal standards. To quantify individual lipid species, the signal intensity ratio of the target compound to its assigned internal standard was multiplied by the added internal standard concentration. Lipid class levels were calculated by summing up all molecular species within each class. For our study, sphingolipid species that had more than 20% of their values missing or that fell below the detection limit in the MIDUS population were excluded from the analysis.

#### Diabetic classification criteria in MIDUS

Patients with or without T2D were categorized based on whether they had a prior medical diagnosis, took prescription medication for diabetes or had fasting blood glucose levels ≥ 126 mg/dL (7.0 mmol/L) or hemoglobin A1c ≥ 6.5% (48 mmol/mol), following the American Diabetes Association criteria [24]. Of the 2,072 participants included, 343 were classified as diabetes cases and 1,729 as non-diabetic controls.

### PREDIMED: study sample and variables

#### Study sample from PREDIMED

Spanish adults included in the case-cohort study were part of the PREDIMED cohort, a primary prevention trial of cardiovascular disease (CVD) carried out from October 2003 through June 2009, as previously described [18, 19]. PREDIMED initially included 7,447 individuals (men aged 55–80 years and women aged 60–80 years) free of CVD, but at high cardiovascular risk. These participants were randomly allocated to three dietary intervention groups: (1) Mediterranean diet supplemented with extra virgin olive oil (MedDiet + EVOO), (2) Mediterranean diet supplemented with mixed nuts (MedDiet + nuts), and (3) a control group advised to follow a low-fat diet.

Among the complete PREDIMED cohort, 3,541 participants were free of T2D at baseline. The current case-cohort study encompasses a random selection of a sample of 692 participants from those who did not have T2D at baseline and with available plasma samples, along with incident T2D cases (n = 250) during a median 3.8-year follow-up. Specifically, the analyzed subcohort included 692 randomly selected subjects (with 53 among them as overlapping cases) and 197 additional T2D cases, totaling 250 cases and 639 non-cases. Additionally, a new measurement was performed on a subset of these participants after 1 year of intervention. Specifically, 646 participants with 1-year follow-up samples and sphingolipid data were analyzed as an internal validation. These included 489 non-cases and 157 cases that were diagnosed after 1 year of follow-up. The selection details are shown in the flow chart available in Fig. S1.

#### Anthropometric, biochemical and clinical variables in PREDIMED

Trained dietitians conducted dual measurements of weight, height, blood pressure and waist circumference. Biochemical variables analyzed in PREDIMED included circulating levels of total cholesterol, LDL-c, HDL-c, triglycerides, and plasma glucose following an overnight fast. These metabolites underwent evaluation utilizing established analytical protocols in accredited biochemistry laboratories. LDL-c was derived through the application of the Friedewald formula. Hypertension was defined as a blood pressure greater than 140/90 mmHg or treatment with antihypertensive medications. Dyslipidemia was defined by LDL-C levels exceeding 4.14 mmol/L (160 mg/dL) or treatment with hypolipidemic agents, and HDL-C concentrations were categorized as dyslipidemic if they were less than 1.29 mmol/L (50 mg/dL) for women or less than 1.03 mmol/L (40 mg/dL) for men, regardless of lipid-lowering therapy.

#### Sphingolipid profiling in PREDIMED

Fasting blood samples collected at baseline and after 1 year of follow-up were processed promptly and frozen plasma stored at -80 °C. Cases and sub-cohort samples were sent to the Broad Institute (Cambridge, MA) for metabolomics assays. Lipid species in plasma were quantitatively profiled using LC-MS, as described elsewhere [25, 26] and detailed in the supplementary material. Detected sphingolipid species included CER16:0, CER22:0, CER24:0, CER24:1, LCER14:0, LCER16:0, and LCER24:1, though CER18:0 could not be quantified. Targeted processing of ceramides and lactosylceramides was conducted using TraceFinder 3.2 software.

#### Adjudication of new cases of T2D

Within the PREDIMED protocol, T2D was established as a predefined secondary endpoint. The Clinical Endpoint Committee, operating in a blinded manner with respect to the intervention groups, oversaw the identification of new instances of T2D during the follow-up period, as detailed elsewhere [18, 27]. Cases were evaluated in accordance with the criteria set forth by the American Diabetes Association [24], which involve either two confirmations of fasting plasma glucose levels ≥ 7.0 mmol/L or 2-hour plasma glucose levels ≥ 11.1 mmol/L subsequent to a 75-g oral glucose challenge.

### Statistical analyses

Participant characteristics were summarized as the mean ± SD for continuous variables or percent occurrence for categorical variables. Student’s t, Wilcoxon, and chi-square tests were performed to assess differences in characteristics between groups. Statistical analyses were conducted using R version 1.2.5001, with RStudio Desktop as the integrated development environment. All analyses were two-tailed and considered statistically significant when *p* ≤ 0.05.

*Volcano plot*. A volcano plot was performed to visualize the overall pattern of sphingolipid species in MIDUS participants with or without T2D. Fold change threshold was set to higher than 1.15 and statistical significance evaluated with the Wilcoxon test. For these analyses, lipid concentrations were used without any transformation.

*Cross-sectional analyses using MIDUS data*. Sphingolipid levels, HOMA-IR, and BMI values were log-transformed to achieve normal data distributions and normalized using z-scores. Associations between sphingolipid species and HOMA-IR were evaluated using linear regression models (adjusting for sex, age, race, sample of origin, BMI, and statin medication), corrected for multiple comparisons and graphed as standardized regression coefficient matrices. Color labels in heat maps represent the regression coefficients (red = positive, and blue = negative). Using the same adjustment variables, the association between high-fat food intake, selected sphingolipid species, and HOMA-IR were evaluated through linear regression models.

Association between sphingolipids and T2D prevalence in MIDUS was assessed using binary logistic regression models adjusted for sex, age, race, sample of origin, BMI and statin medication. Regression effects were presented as odds ratios (OR) with 95% confidence intervals (CI). When generating sphingolipid biomarkers (sum of selected lactosylceramide species or the ratio to CER22:0; see Results), calculations were performed prior to log transformation. Age was treated as a continuous variable, educational attainment was categorized into 3 levels: (1) high school or less, (2) college, and (3) postgraduate studies. Race was coded as white and others.

*Longitudinal analysis using the PREDIMED data*. To assess the prospective associations of the sphingolipid species with the incidence of T2D, 3 lactosylceramides (LCER14:0, 16:0 and 24:1) and 1 ceramide (CER22:0) species were selected based on their association with T2D in MIDUS and their availability in PREDIMED. In addition, the sum of three selected lactosylceramide species as well as their ratio to CER22:0 levels were calculated and used as composite scores. Subsequently, these values were normalized using the Blom’s rank-based inverse transformation [28]. Weighted proportional hazards Cox regression models [29] with Barlow weights were used to estimate HRs and their 95% CIs, using sphingolipid species concentrations and scores as continuous variables or categorized into quartiles. Follow-up time was calculated from the date of enrollment to the date of T2D diagnosis for cases, and to the date of the last visit or the end of the follow-up period for non-cases. Model I was adjusted for age, sex, BMI level, and intervention group. Model II was additionally adjusted for hypertension, dyslipidemia, and smoking. And model III included all the mentioned variables, in addition to basal glycemia. Cumulative incidence curves were estimated with the Makuch-Ghali method from Cox regressions [30]. Absolute risks and risk differences at 5 years were obtained from the cumulative incidences. Confidence intervals were obtained from bootstrap distributions (B = 500).

For internal validation, the same models (I, II and III) were used to assess the association between 1-year plasma sphingolipid levels with the risk of subsequent T2D using only the T2D cases that occurred after 1-year follow-up. For the comparison between extreme quartiles, these were calculated independently at each time point based on the sphingolipid levels of the respective population. Additionally, given the hypothesis-driven nature of the analysis and to prioritize the reduction of type II error, corrections for multiple comparisons were not applied.

## Results

### Characterization of sociodemographic and clinical variables in MIDUS

A descriptive summary of sociodemographic and health variables for MIDUS participants without and with T2D are provided in Table 1. Individuals with T2D were more likely to be older and to present a higher percentage of non-white participants. They exhibited higher BMI and waist circumference, more atherogenic dyslipidemia (high levels of triglycerides and low levels of HDL-c) and unhealthy glucoregulatory indices as compared to those without T2D. Moreover, these patients had significantly higher statin use and lower levels of LDL-c as compared to those without T2D.

**Table 1:** Sociodemographic and health description of MIDUS participants with and without T2D

	Without T2D n = 1729	With T2D n = 343
Sex (% females)	54.8	54.5
Race (% whites)	78.1	58.0
Statin use (%)	21.3	41.7
Age (years-old)	54.8 ± 12.6	59.6 ± 11.4
Body mass index (kg/m^2^)	29.1 ± 6.3	34.2 ± 8.4
Waist circumference (cm)	95.7 ± 16.7	110 ± 19.2
LDL-cholesterol (mg/dL)	104.1 ± 34.3	95 ± 38.5
HDL-cholesterol (mg/dL)	58.0 ± 18.7	50.7 ± 17.7
Total cholesterol (mg/dL)	186.1 ± 38.7	177.0 ± 46.0
Triglycerides (mg/dL)	119.5 ± 87.7	161.2 ± 191.2
HbA1c (%)	5.6 ± 0.4	7.6 ± 1.9
Glycemia (mg/dL)	95.3 ± 9.7	135.7 ± 52.6
Insulin (mIU/mL)	13.3 ± 12.4	25.9 ± 29.6
HOMA-IR	3.2 ± 3.1	8.7 ± 11.1

All variables were significantly different between both groups, except for sex

### Sphingolipids associated with insulin resistance and prevalence of T2D in MIDUS

A volcano plot was performed to visualize the overall pattern of sphingolipid species in MIDUS participants with or without T2D (Fig. S2). Most ceramide and dihydroceramide species were elevated in individuals with T2D, whereas most lactosylceramide and hexosylceramide species were reduced. Only a few species of sphingomyelins showed differences between individuals with and without T2D, all of which exhibited mild changes. Based on these results, subsequent analyses focused on ceramides, dihydroceramides, hexosylceramides, and lactosylceramides.

**Fig. 1:**
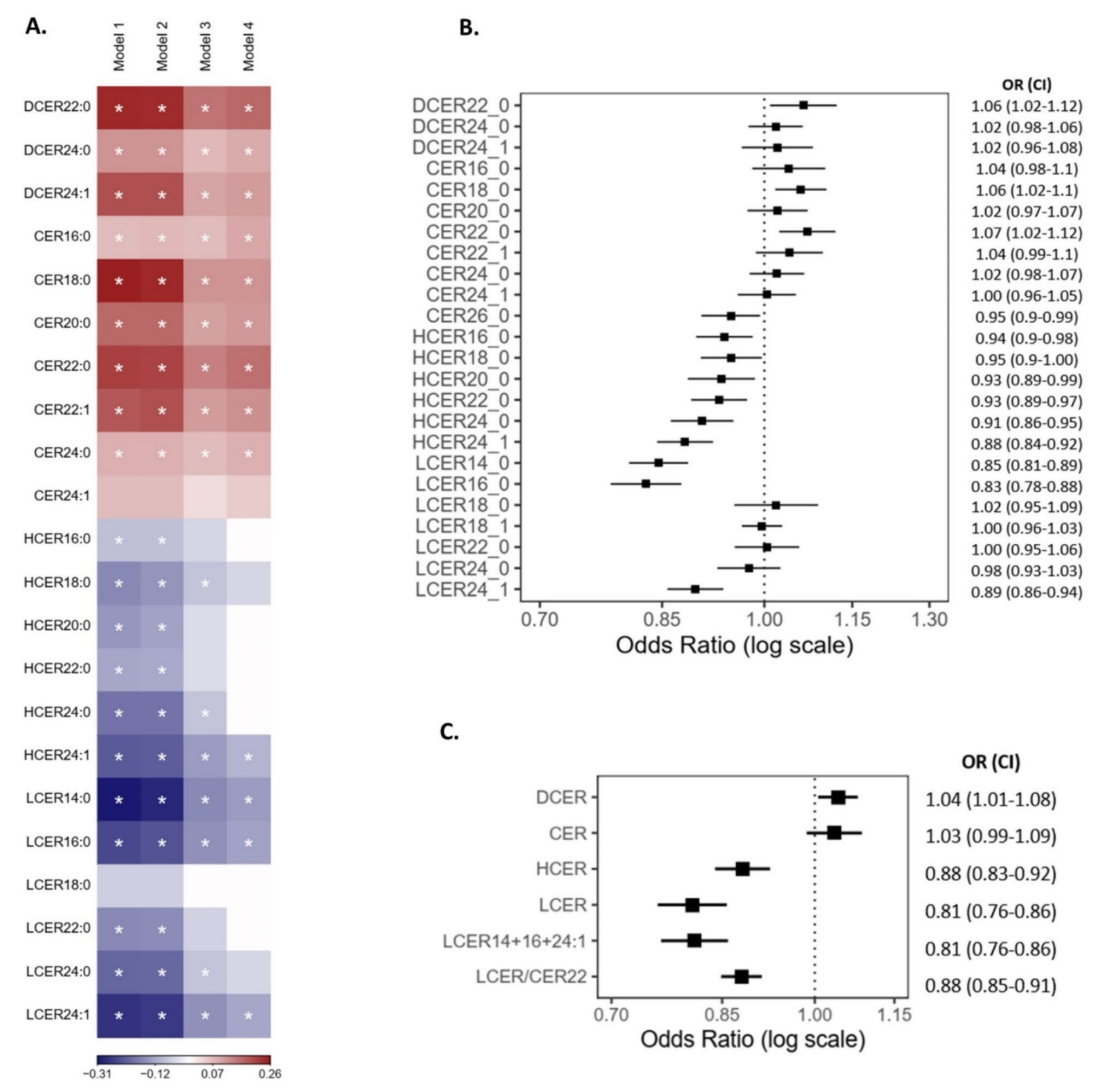
Association of blood sphingolipid levels, insulin resistance and T2D prevalence in MIDUS. **A** Linear regression model for the association between sphingolipid levels and HOMA-IR. Model 1: bivariate, Model 2: adjusted for sample of origin, race, sex, and age, Model 3: Model 2 plus BMI; and a Model 4: Model 3 plus statin use. Color label represents the standardized regression coefficient (positive in red, and negative in blue) of those associations. Asterisks denote significant associations with a Bonferroni-adjusted *p* < 0.05.** B** Odds ratios for T2D (with 95%CI) per SD in blood levels of sphingolipid species, adjusted for sample of origin, race, sex, age, BMI, and statin use.** C** Odds ratios for T2D (with 95%CI) per increase in serum levels of sphingolipid classes as well as a set of selected species and LCER/CER22 ratio, adjusting for sample, race, sex, age, BMI, and statin use

To identify species potentially associated with the etiology and pathophysiology of T2D, regression models were used to assess the relationship between sphingolipid species, insulin resistance (HOMA-IR), and T2D prevalence. All these analyses were adjusted for sociodemographic variables, sample of origin, BMI, and statin use. As shown Models I and II (bivariate and partially adjusted, respectively) in Fig. 1A, serum levels of most dihydroceramide and ceramide species were associated with higher insulin resistance, whereas opposite relationships were observed for hexosylceramide and lactosylceramide species. After adjusting for major potential confounders, only the positive associations of some dihydroceramide and ceramide species as well as the negative associations of certain lactosylceramide species with HOMA-IR remained statistically significant. The lipid species exhibiting the strongest direct associations were DCER22:0 and CER22:0 (Coefficient [CI95%] = 0.17 [0.13, 0.21] and 0.16 [0.12, 0.20], respectively), whereas the species with the clearest inverse associations were LCER14:0, LCER16:0, and LCER24:1 (Coefficient [CI95%] = -0.15 [-0.19, -0.11], -0,14 [-0.18, -0.11], and − 0.14 [-0.17, -0.10], respectively). The coefficients of the aforementioned models and their adjusted *p*-values are presented in Table S1. To assess the impact of high-saturated-fat food intake on this relationship, we evaluated the association between the consumption of high-fat meats and fast food with levels of the highlighted lipids, as well as the potential confounding effect of these variables on the association between sphingolipid species and insulin resistance. As shown in Table S2, high-saturated-fat food intake did not demonstrate a significant association with most sphingolipid species, and after including these variables as adjustment factors, the association between the sphingolipid species and HOMA-IR remained significant.

When assessing the association of sphingolipid species with T2D, only direct associations with T2D were observed for DCER22:0, CER18:0, and CER22:0 in the fully adjusted models (Fig. 1B). Further, most glycosphingolipid species exhibited a statistically significant inverse association with T2D. The most pronounced inverse associations with T2D prevalence were observed for LCER14:0, LCER16:0, and LCER24:1. These cross-sectional analyses were repeated using lipid classes ‒the summed concentrations of all identified species within the same class‒ or composite scores, consider whether they provided more sensitive and robust bioindicators. In particular, LCER14:0, LCER16:0, LCER24:1 were selected because of their strong negative association with HOMA-IR and T2D prevalence. As shown in Fig. 1C, higher dihydroceramide levels were significantly associated with 4% higher odds of T2D (OR = 1.04, 95% CI: 1.01–1.08), while the ceramides class showed no association. Regarding lactosylceramides, both the total class and the sum of the selected species were associated with a significantly 19% relatively lower prevalence of T2D. To mitigate the interindividual variability in total sphingolipid levels, a score was calculated using a ratio between the sum of the selected lactosylceramides in the numerator and CER22:0 in the denominator, because it was the ceramide species previously found to exhibit the strongest association with HOMA-IR and T2D prevalence. This ratio was also significantly associated with a 12% relatively lower prevalence of T2D.

### Characterization of sociodemographic and clinical variables in PREDIMED

A longitudinal analysis was conducted to assess if the species associated with insulin resistance and T2D in the MIDUS study could also predict the incidence of T2D in the PREDIMED study. Baseline descriptions of PREDIMED participants from the subcohort, as well as comparisons between baseline variables of non-cases and new T2D cases, are provided in Table 2.

**Table 2:** Baseline demographic and health description of the PREDIMED participants without and with T2D at follow-up

	Complete subcohort	Non-cases (n = 639)	T2D cases (n = 250)	*p*-value
Sex, females, n (%)	546 (61.4)	408 (63.8)	138 (55.2)	*
Age (years-old)	66.5 ± 5.8	66.6 ± 5.8	66.4 ± 5.7	ns
Body mass index (kg/m^2^)	30.1 ± 3.5	29.8 ± 3.6	30.8 ± 3.3	***
LDL-cholesterol (mg/dL)	140.3 ± 30.7	140.3 ± 33.7	136.1 ± 33.0	ns
HDL-cholesterol (mg/dL)	56.1 ± 15.0	57.6 ± 15.7	52.7 ± 12.4	***
Total cholesterol (mg/dL)	221.1 ± 39.3	221.8 ± 38.3	219.7 ± 41.7	ns
Triglycerides (mg/dL)	137.5 ± 81.7	127.0 ± 63.0	163.6 ± 111.6	***
Glycemia (mg/dL)	103.4 ± 17.2	98.0 ± 13.6	117.1 ± 17.6	***
Dietary intervention				ns
MedDiet + EVOO, n (%)	272 (30.6)	198 (31)	74 (29.6)	
MedDiet + NUTS, n (%)	322 (36.2)	237 (37.1)	85 (34)	
LowFatDiet, n (%)	295 (33.2)	204 (31.9)	91 (36.4)	

Complete sub-cohort (N = 889) includes the selected sub-cohort (N = 692) plus the additional 117 cases. MedDiet + EVOO and MedDiet + NUTS: Mediterranean diet supplemented with extra virgin olive oil or nuts. *p < 0.05 and ***p < 0.001 when comparing frequencies by Chi-square or means by t-test

### Lactosylceramides associated with incidence of T2D in PREDIMED

Association analyses were performed in PREDIMED between baseline levels of sphingolipid species and incident cases of T2D during a median follow-up of 3.8 years, adjusting for age, sex, BMI, recruitment center and intervention group (Model I). Subsequently, adjustment was made additionally for the prevalence of dyslipidemia, hypertension, and smoking (Model II). Weighted Cox-regression models were conducted using the levels of each lipid species individually, summing the selected lactosylceramide species (LCER14:0, 16:0, and 24:1), and using the ratio between selected lactosylceramides and CER22:0 levels.

**Fig. 2:**
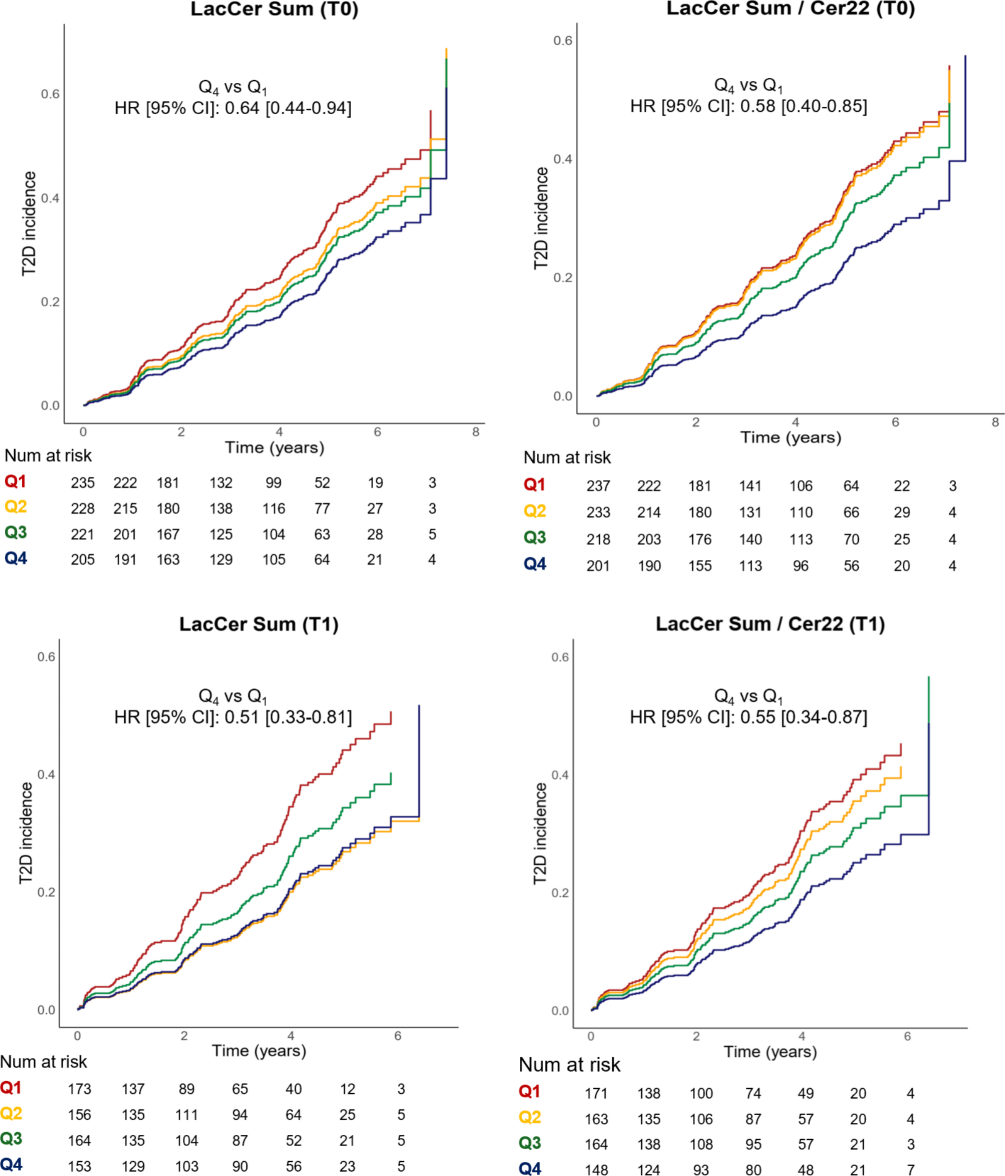
Estimates of the cumulative incidence of T2D based on quartiles of selected sphingolipid species in PREDIMED. Quartiles of** A** baseline levels (T0) of the sum of selected LacCer species (LCER14:0, LCER16:0, and LCER24:1),** B** baseline levels (T0) of the ratio of the selected lactosylceramides to CER22:0,** C** levels of the selected LacCer after one year of intervention (T1), and** D** levels of the ratio of these LacCer to Cer22:0 after one year of intervention (T1). Hazard Ratio (95% CI) for quartile 4 (highest concentration of LacCer) compared to quartile 1 (reference) and the *p*-value are shown. In both cases, Cox regression was performed, adjusting for age, sex, BMI level, intervention group, and recruitment center

When evaluated individually, the trends were similar to those observed in MIDUS; but none of the species showed a significant association with the incidence of T2D (Table S3). However, as shown in Fig. 2A, individuals in the upper quartile of the sum of lactosylceramide species exhibited a significantly lower incidence of T2D as compared to those in the lowest quartile of these species (HR_Q4vsQ1_=0.64, 95% CI 0.44–0.94, *p* = 0.024 for Model I). Similarly, the inverse association between the ratio of lactosylceramides to CER22:0 and T2D incidence showed a stronger significance (Fig. 2B). The upper quartile of this index was associated with a lower risk of T2D when comparing it with the lowest quartile (HR_Q4vsQ1_=0.58, 95% CI 0.39–0.85, *p* = 0.0016). Interestingly, this ratio enabled a more effective quartile stratification, leading to a significant* p*-value for trend (*p* = 0.014), unlike the sum of lactosylceramides alone (*p* = 0.053).

At 5-years of follow-up, the absolute risk of T2D for the lactosylceramide sum was 9.6% (95% CI 0.3%-20.5%, *p* = 0.044) lower for Q4 (26.9%, 95%CI 19.6-34.6%) than Q1 (36.6%, 95% CI 29.4%-44.0%). Similarly, for the ratio of lactosylceramides to CER22:0, the absolute risk was 11.4% (95% CI 1.0%-21.6%, *p* = 0.036) lower for Q4 (25.0%, 95%CI 18.3-32.6%) vs. Q1 (36.4%, 95% CI 28.7%-44.4%).

Both scores also showed a statistically significant association with the incidence of T2D when used as continuous variables (Table 3). Furthermore, this association persisted for both quartile comparisons and continuous variables after adjusting for hypertension, dyslipidemia, and smoking (Table 3, Model II). Significance was only lost when adjusting for fasting glucose levels at baseline (Table 3, Model III), the main predictor variable for diabetes. The correlation between levels of selected sphingolipid species and each of the covariates used, including fasting glycemia, is presented in Table S4.

**Table 3:** Estimates of T2D risk based on selected sphingolipid levels in PREDIMED

Time	Predictor as continuous variable	Model I		Model II		Model III	
		HR (95% CI)	*p*-value	HR (95% CI)	*p*-value	HR (95% CI)	*p*-value
*T0*	LCER14:0 + LCER16:0 + LCER24:1	0.85 (0.72–0.99)	0.040	0.85 (0.72–0.99)	0.049	0.88 (0.71–1.01)	0.203
*T0*	(LCER14:0 + LCER16:0 + LCER24:1) / CER22:0	0.75 (0.64–0.89)	0.001	0.79 (0.66–0.92)	0.003	0.85 (0.69–1.05)	0.135
*T1*	LCER14:0 + LCER16:0 + LCER24:1	0.79 (0.65–0.97)	0.021	0.81 (0.66–0.99)	0.042	0.79 (0.61–1.03)	0.083
*T1*	(LCER14:0 + LCER16:0 + LCER24:1) / CER22:0	0.76 (0.61–0.94)	0.010	0.80 (0.65–0.98)	0.034	0.81 (0.62–1.07)	0.147

Time	Predictor (Q_4_vs Q1)	Model I		Model II		Model III	
		HR (95% CI)	*p*-value	HR (95% CI)	*p*-value	HR (95% CI)	*p*-value
*T0*	LCER14:0 + LCER16:0 + LCER24:1	0.64 (0.44–0.94)	0.024	0.66 (0.46–0.97)	0.032	0.92 (0.51–1.66)	0.785
*T0*	(LCER14:0 + LCER16:0 + LCER24:1) / CER22:0	0.58 (0.40–0.85)	0.006	0.63 (0.43–0.92)	0.018	0.57 (0.29–1.08)	0.090
*T1*	LCER14:0 + LCER16:0 + LCER24:1	0.51 (0.33–0.81)	0.004	0.53 (0.33–0.83)	0.006	0.58 (0.29–1.15)	0.119
*T1*	(LCER14:0 + LCER16:0 + LCER24:1) / CER22:0	0.55 (0.34–0.87)	0.013	0.65 (0.41–1.04)	0.07	0.74 (0.35–1.51)	0.404

Model I: Age, sex, recruitment center, intervention group and BMI level

Model II: Age, sex, recruitment center, intervention group, BMI level, dyslipidemia, hypertension, and smoking

Model III: Age, sex, recruitment center, intervention group, BMI level, dyslipidemia, hypertension, smoking and fasting glycemia

For internal validation, these analyses were repeated using sphingolipid levels measured after one year of follow-up in PREDIMED. As shown in Fig. 2C (Model I), higher levels of these lactosylceramides after one year were associated with a lower risk of T2D incidence. Again, this association was statistically significant when comparing extreme quartiles (HR_Q4vsQ1_=0.51, 95% CI 0.33–0.81, *p* = 0.004) or when using the sum of these species as a continuous variable (Table 3, Model I), and even after additionally adjusting for prevalence of hypertension, dyslipidemia and smoking (Table 3, Model II). A significant inverse association with the incidence of T2D was also observed for the ratio between these lactosylceramides and CER22:0 after one year of nutritional intervention, both when comparing extreme quartiles and when using it as a continuous variable (Table 3, Model I). Again, this ratio -measured after one year of follow-up- allowed for better quartile stratification, leading to a significant *p*-value for trend (*p* = 0.024), unlike the sum of lactosylceramides alone (*p* = 0.06). The 5-year absolute risk of T2D for the lactosylceramide sum was 14.3% lower (95%CI 92.0%-28.0%, *p* = 0.048) lower for Q4 vs. Q1. The second score deemed a risk difference of 13.4% (95% CI 0.5%-26.7%, *p* = 0.032), lower for Q4 vs. Q1. Finally, when adjusting for the prevalence of comorbidities, the association remained significant for the ratio as a continuous variable, but not for the comparison between extreme quartiles (Table 3, Model II).

To evaluate whether these sphingolipid scores could differentiate the risk among the prediabetic population, the same models (I, II, and III) were repeated in subjects with baseline glucose levels above 100 mg/dL (Table S5). According to our results, both sphingolipid scores were associated with a lower risk of T2D, even after adjusting for comorbidities. In all cases, significance was lost when adjusting for fasting glucose, suggesting that their predictive power may be partially mediated by glycemic status.

## Discussion

The findings from this analysis of two large cohort studies in the United States and Spain revealed significant inverse association between blood levels of lactosylceramides and alterations in glucose metabolism. We were able to identify three species of lactosylceramide (LCER 14:0, 16:0, and 24:1) that exhibited a strong inverse relationship with both insulin resistance and the prevalence of diabetes in the American population. In contrast, some ceramides were linked to increased insulin resistance and higher risk of T2D. Particularly, only CER18:0 and CER22:0 were additionally associated with a higher prevalence of diabetes. Based on this information, two Lactosylceramide- and Ceramide-based scores were created, which were externally validated in the Spanish population of PREDIMED also with repeated measurements after 1-year follow up, and they consistently showed an inverse association with the incidence of T2D.

Ceramides have been implicated in the development of insulin resistance and beta-cell dysfunction [7, 31]. Elevated ceramide levels have been observed in skeletal muscle and adipose tissue of individuals with diabetes [32, 33]. Moreover, some ceramide species appear to disrupt insulin signaling pathways and promote inflammation, contributing to impaired glucose homeostasis (reviewed in [34]). In this context, researchers have postulated that other classes of ceramides-derived glycosphingolipids, including lactosylceramides, may contribute to impaired glucose homeostasis. This hypothesis primarily stems from observations made in different murine models of diabetes, where the accumulation of lactosylceramide species has been identified in skeletal muscle and the heart [35, 36]. This accumulation may contribute to mitochondrial dysfunction, oxidative stress, and inflammation [16, 36], with potential pathogenic roles in diabetes. In addition, a study demonstrated that inhibiting the synthesis of lactosylceramides, and all downstream glycosphingolipids, improved the metabolic status of db/db diabetic mice [37]. Nevertheless, it is our contention that in humans the role of lactosylceramides in glucose metabolism, as well as in the development of diabetes, remains uncertain. While one study observed a direct association of lactosylceramide species with future cardiovascular outcomes in diabetic patients [12], to the best of our knowledge, no study has found a positive association of these lipids with the incidence of new cases of diabetes. In fact, lipidomic studies in different populations have reported associations between lactosylceramides and improved glucose metabolism indicators or lower diabetes incidence [14, 21]. In this context, our study provides compelling evidence regarding the inverse association between plasma levels of LCER14, 16, and 24:1 and alterations in glucose metabolism and the development of diabetes in two distinct populations.

The mechanism underlying the inverse relationship between lactosylceramides and diabetes remains uncertain. It could reflect an indirect association resulting from an upregulation of the salvage pathway, leading to higher ceramide production via the catabolism of glycosphingolipids [21, 38]. Similarly, it could also result from increased turnover for enhanced GM3 formation, another glycosphingolipid associated with diabetes [39]. On the other hand, lactosylceramides fulfill most of their functions by structuring glycosphingolipid-enriched microdomains in cell membranes and could be involved in glucose homeostasis signaling pathways. For instance, the induction of glucosylceramide and lactosylceramide synthesis in myotubes has been shown to enhance insulin signaling [40]. It is important to note that including fasting glucose in the model reduced the independent predictive power of the lipid biomarkers, suggesting that while sphingolipids are valuable predictors of T2D, their effects may overlap with or be mediated by glucose metabolism. Considering our results and the available evidence, it appears that glycosphingolipids levels decrease in parallel with metabolic alterations, such as insulin resistance and metabolic syndrome, possibly due to an increase in the salvage pathway [21, 38]. It is possible that once these metabolic alterations are established, the salvage pathway may no longer compensate, and the excessive increase of some lactosylceramide species, along with ceramide levels, induces inflammation, oxidative stress and further increases cardiovascular risk [16]. This could explain the associations observed by other researchers between high levels of lactosylceramides and increased cardiovascular risk [12, 13].

Regarding ceramides, while the majority of their species were associated with insulin resistance, only CER 18:0 and 22:0 species were significantly correlated with the prevalence of diabetes in the American population of MIDUS after all adjustments. Both have been identified previously as potential diabetes biomarkers [41]. In our longitudinal evaluation of the Spanish PREDIMED cohort, CER18:0 was not included in the lipid panel, but we did have CER22:0 levels. While the levels of this species alone were not associated with a higher incidence of diabetes, when used as the denominator in the lactosylceramide/ceramide score, it did improve the risk stratification of the score quartiles. These results support the hypothesis of increased activity in the ceramide salvage pathway in these subjects. Even though this study does not demonstrate such mechanism, the scores reported here could serve as valuable novel biomarkers of glucose metabolism abnormalities and incidence of diabetes cases. Moreover, a recent study found differences in the levels of circulating sphingolipids between patients with T2D and T1D [42]. Patients with T2D exhibited increased levels of various ceramide species, whereas patients with T1D did not. Conversely, certain species of lactosylceramides were found to be decreased in individuals with T2D but increased in subjects with T1D. The observed results by this group are consistent with our findings and suggest that our risk prediction may be specific to T2D.

An important strength of this study lies in its inclusion of two large, ethnically and culturally diverse populations, one from the United States and the other from Spain. Despite the inherent variations in lifestyle, genetics, and cultural practices, the study reveals consistently significant results. Moreover, by conducting a comprehensive lipidomic analysis, our work moved beyond mere class identification and delved into the specific species of sphingolipids that were implicated in both populations.

This study offers the advantage of not only encompassing a cross-sectional analysis in the American population but also a longitudinal analysis in a sizable Spanish population with high cardiometabolic risk, spanning over a period of more than 4 years. In addition, the inclusion of both baseline and one-year follow-up measurements of sphingolipids in the Spanish sample further allows for internal validation of the results. The follow-up analysis was conducted at different time points, specifically after one year of lifestyle changes during which certain lipid levels fluctuated. Despite these changes, the selected biomarkers continued to accurately predict the risk of T2D, thus reinforcing their robustness.

One limitation of this study is its observational nature, which reduces the possibility of establishing causal relationships. In addition, while the evaluation of circulating sphingolipids is well-suited for identifying systemic biomarkers, it may not directly reflect the actual sphingolipids concentrations in specific target tissues, making it challenging to draw underlying mechanistic conclusions. Moreover, there may still be unmeasured confounding variables, as the lack of CER18:0 levels in the PREDIMED study. In addition, some medications currently used in patients with T2D may help improve the sphingolipid profile. Several studies have reported that GLP-1 agonists can reduce certain ceramide species associated with cardiovascular risk [43, 44]. However, the potential effect on the lactosylceramide profile remains to be investigated. This study includes populations examined prior to 2009, when the use of these agonists was very limited. It was with the approval of long-acting GLP-1 agonists, such as liraglutide in 2010, that this therapeutic class began gaining wider acceptance in the treatment of T2D.

Finally, due to high inter-laboratory variability, it is not advisable to compare absolute concentration values of these analytes between different samples. Therefore, careful consideration of experimental procedures is required.

Nevertheless, considering the robust inverse association of lactosylceramide score with insulin resistance and T2D in both populations, reduced levels of these lactosylceramides, specially with high levels of CER22:0, could potentially serve as valuable indicators of disruption in glucose regulation and their progression to T2D. It is crucial to elucidate the involved mechanisms, determine if a cause-and-effect relationship exists, and assess whether these lipids or their regulatory enzymes could serve as lifestyle and/or pharmacological targets. However, we underscore the need for prudence when considering the use of inhibitors for glycosphingolipid synthesis as a way to improve metabolic health, especially in the absence of a thorough comprehension of the function of lactosylceramides in glucose metabolism.

## Conclusions

This study revealed a significant inverse association between specific lactosylceramide species (LCER 14:0, 16:0, and 24:1) and glucose metabolism alterations, as well as a lower risk of T2D in both the American and Spanish populations studied. Conversely, certain ceramides, notably CER22:0, was linked to increased insulin resistance and a higher prevalence of T2D. These findings suggest that reduced levels of lactosylceramide species, particularly in combination with high CER22:0 levels, could serve as valuable biomarkers for identifying disruptions in glucose regulation and predicting T2D progression. However, further studies are required to explore the underlying mechanisms, determine causality, and assess the potential of these lipids or their regulatory enzymes as therapeutic targets.

## Electronic supplementary material

Below is the link to the electronic supplementary material.

Supplementary Material 1.

## Data Availability

MIDUS data can be found here: https://midus.colectica.org/. Lipidomic data was deposited to OSF database under the https://doi.org/10.17605/OSF.IO/VFR7B. Requestors wishing to access the PREDIMED data used in this study can request it to the PREDIMED Steering Committee: predimed-steering-committe@googlegroups.com.

## Abbreviations

BMI: Body mass index
CER: Ceramide
CI: Confidence interval
CVD: Cardiovascular disease
HbA1c: Hemoglobin A1c
HDL-c: High density lipoprotein cholesterol
HOMA-IR: Homeostatic model assessment of insulin resistance
HR: Hazard ratio
LC-MS: Liquid chromatography-mass spectrometry
LCER: Lactosylceramide
LDL-c: Low density lipoprotein cholesterol
MIDUS: Midlife in the United States’ study
OR: Odds ratio
PREDIMED: Prevención con Dieta Mediterránea’ study
Q1: First l
Q4: Fourth quartile
T2D: Type 2 diabetes
