# Supplementary material for: Sphingolipid profiling as a biomarker of type 2 diabetes risk: evidence from the MIDUS and PREDIMED studies
Source: Cardiovasc Diabetol. 2024 Dec 18;23:446. doi: 10.1186/s12933-024-02505-7 (PMC11657495; doi:10.1186/s12933-024-02505-7)
Supplement: Supplementary file 1 — Supplementary Material 1. [file 12933_2024_2505_MOESM1_ESM.pdf]

Sphingolipid profiling as a biomarker of Type 2 Diabetes risk: Evidence from the MIDUS and PREDIMED studies

Loni Berkowitz[1], Cristina Razquin[2], Cristian Salazar[1], Fiorella Biancardi[1], RamónEstruch[3,4], Emilio Ros[5], Montserrat Fitó[6], Dolores Corella[4,6], Christopher L Coe[7], Carol D Ryff[8], Miguel Ruiz-Canela[2], Jordi Salas-Salvado[6,9,10], Daniel Wang[11], Frank B. Hu[11,12], Amy Delk[13], Miguel Ángel Martínez-Gonzalez[2], Attilio Rigotti[1].

Supplementary Material

1. Sphingolipid profiling in MIDUS.
2. Sphingolipid profiling in PREDIMED.
3. Fig S1. Flowchart of Patient Selection in the PREDIMED Cohort.
4. Fig S2. Volcano plots of sphingolipid species levels in adults with or without T2D.
5. Table S1. Standardized regression coefficients of those associations between sphingolipids and HOMA-IR in MIDUS.
6. Table S2. Standardized regression coefficients of those associations between sphingolipid levels, HOMA-IR, and intake of saturated fat-rich foods.
7. Table S3. Estimates of T2D risk based on levels of selected sphingolipid species in PREDIMED.
8. Table S4. Correlation between selected lipid levels and confounders used in PREDIMED.
9. Table S5. Estimates of T2D risk based on selected sphingolipid levels in prediabetic patients form PREDIMED.

Sphingolipid profiling in MIDUS

Lipid profiling was performed as part of an untargeted-lipidomic approach by Metabolon, Inc. (Durham, NC), following the protocol described below.

- Serum Sample Collection: Blood was drawn by clinical phlebotomists after an overnight fast, in the early morning upon participants' awakening. All participants had spent the night at one of three Clinical and Translational Research Centers (CTRCs). Blood samples were centrifuged in a refrigerated centrifuge by CTRC nurses on the morning of collection, with the serum fraction frozen in 0.5 mL aliquots and stored in cryovials in an ultracold freezer at -70°C. Previously unthawed sera were then shipped overnight on dry ice for LC/MS metabolomic analysis (Metabolon, Morrisville, NC). High-throughput processing ensured that all samples were analyzed within the same run, generating analyte identifications and concentrations in a consistent manner.

- Extraction: Lipids were extracted from the bio-fluid in the presence of deuterated internal standards using an automated BUME extraction according to the method of Lofgren et al. 2012 (DOI: 10.1194/jlr.D023036).

- Data Acquisition: The extracts were dried under nitrogen and reconstituted in ammonium acetate dichloromethane:methanol. The extracts were transferred to vials for infusion-MS analysis, performed on a Shimadzu LC with nano PEEK tubing and the Sciex SelexIon-5500 QTRAP. The samples were analyzed via both positive and negative mode electrospray. The 5500 QTRAP was operated in multiple reaction monitoring (MRM) mode with a total of more than 1,100 MRMs.

- Quantification: Quantitation was built using class-specific internal standards with each class containing at least one, with most containing multiple labeled internal standards. Individual lipid species were quantified by taking the ratio of the signal intensity of each target compound to that of its assigned internal standard, then multiplying by the concentration of internal standard added to the sample (e.g. dCER (d16:0) and dDCER(16:0) for ceramides and dihydroceramides, respectively). Lipid class concentrations were calculated from the sum of all molecular species within a class, and fatty acid compositions were determined by calculating the proportion of each class comprised by individual fatty acids. The quantification of the different species and classes of lipids is reported as micromolar concentration. For our study, we only used sphingolipid values (dihydroceramides, ceramides, sphingomyelins, hexosylceramides -encompassing glucosylceramides and galactosylceramides, and lactosylceramides).

- Data processing: Before statistical analyses, sphingolipid levels were loge-transformed to achieve normal distributions and normalized using z-score. Only those species with <20% of missing values (due to levels below the lower limit of detection) were included in the statistical modeling

Sphingolipid profiling in PREDIMED

- Fasting blood samples collected at baseline and after 1 year of follow-up were processed promptly and frozen plasma stored at -80°C. Cases and sub-cohort samples were sent to the Broad Institute (Cambridge, MA) for metabolomics assays where lipid species in plasma were quantitatively profiled using LC-MS.

- The internal standard, 1,2-didodecanoyl-sn-glycero-3-phosphocholine, was included as a quality control measure to ensure proper injection of each sample into the LC-MS system and to monitor for potential changes in MS sensitivity over the analysis period. During initial setup and method validation, seventy standards for sphingomyelins were analyzed, and pooled reference samples were used to correct for any instrument drift. Specifically, a pooled reference sample, created from study samples, was injected every 20 samples, in duplicate. One of these pools was used to correct for MS sensitivity drift, while the second pool was employed to calculate the coefficient of variation and assess reproducibility for each feature.

- Individual lipid abundances were measured as integrated LC-MS peak areas (unitless) and standardized relative to pooled plasma samples using a "nearest neighbor" approach, helping mitigate minor MS drift effects both within and between sample batches. Lipid species were identified based on fatty acyl carbon content, saturation levels, parent ion mass-to-charge ratios, and retention time patterns for each lipid class. While standards for every individual lipid are not commercially available, representative standards informed identifications through characteristic features in MS/MS product ion spectra.

- For lactosylceramides, we utilized six reference compounds from Avanti Polar Lipids and Sigma-Aldrich: LacCer(d18:1/8:0), LacCer(d18:1/12:0), LacCer(d18:1/16:0), LacCer(d18:1/17:0), LacCer(d18:1/18:0), and LacCer(d18:1/24:0).

- Targeted processing of ceramides and lactosylceramides was conducted using TraceFinder 3.2 software.

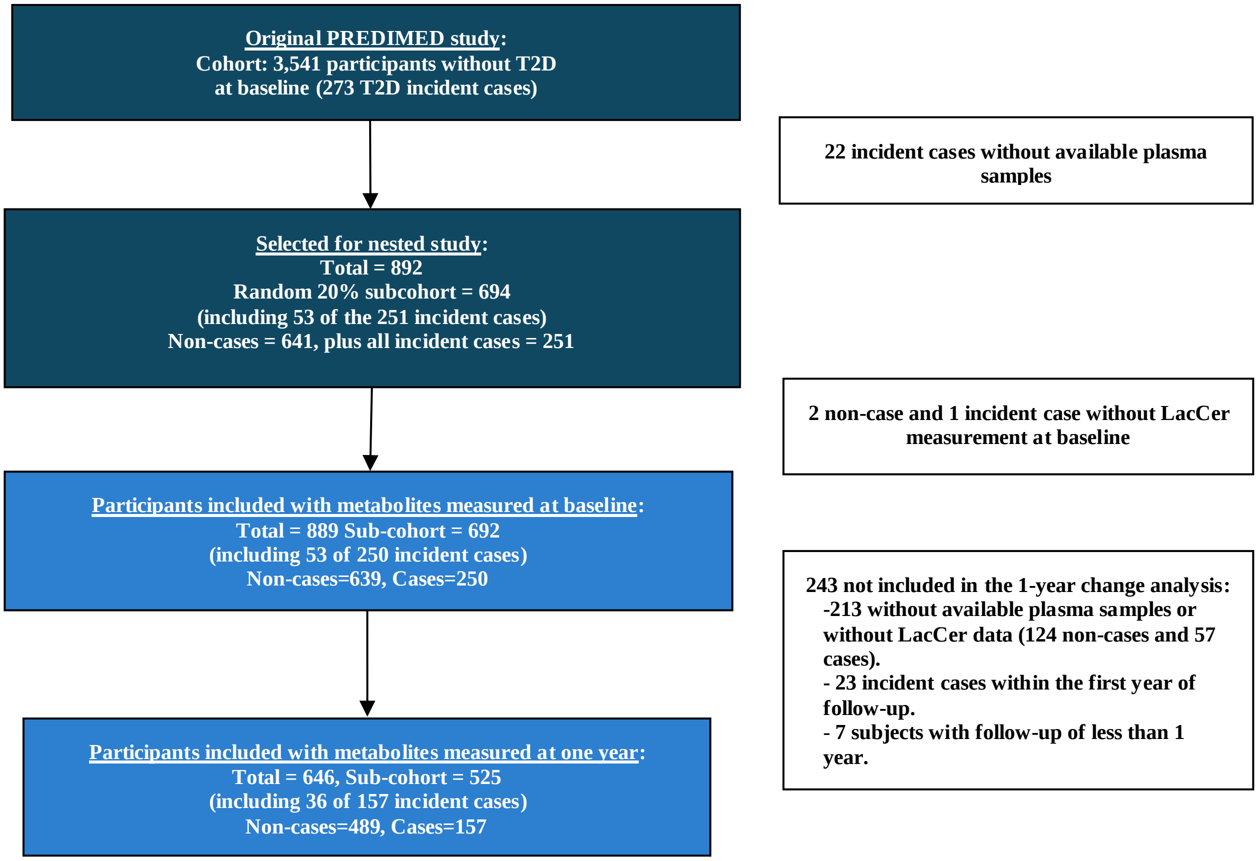

Fig S1. Flowchart of Patient Selection in the PREDIMED Cohort

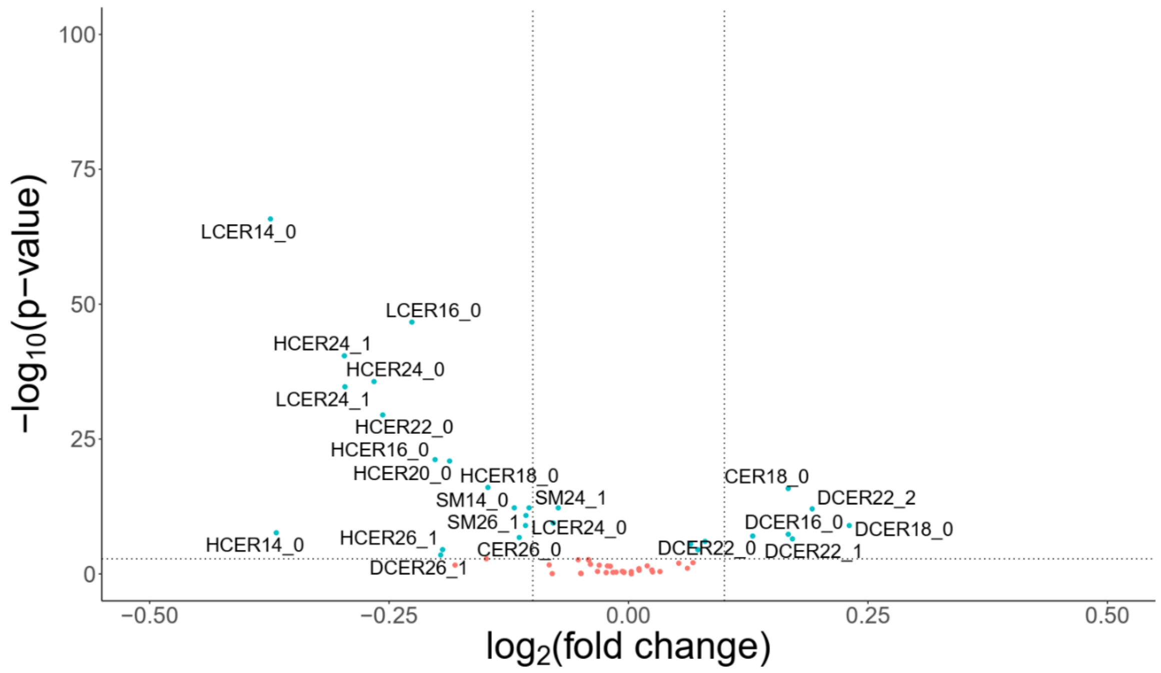

Fig S2. Volcano plots of sphingolipid species levels in adults with or without T2D. Statistical significance was evaluated with the Wilcoxon-test and fold the change threshold in sphingolipids was set to higher than 1.15. Negative and positive and $\log_2$(fold change) indicates decreased or increased sphingolipid abundance in adults with or without T2D. Light blue circles denote p-value < 0.05. Raw data were used in this analysis.

Table S1. Standardized regression coefficients of those associations between sphingolipids and HOMA-IR in MIDUS.

	Model 1	*Model 2*	*Model 3*	*Model 4*
	Coef (p-value)	***Coef (p-value)***	***Coef (p-value)***	***Coef (p-value)***
**DCER22:0**	0.26 (3.1E-26)	0.25 (9.9E-26)	0.17 (1.1E-15)	0.17 (1.3E-15)
**DCER24:0**	0.13 (9.3E-07)	0.13 (1.3E-06)	0.08 (5.6E-04)	0.09 (1.7E-04)
**DCER24:1**	0.21 (6.3E-19)	0.21 (3.1E-19)	0.11 (8.2E-07)	0.11 (1.9E-06)
**CER16:0**	0.08 (2.3E-02)	0.08 (1.6E-02)	0.08 (1.3E-03)	0.08 (1.3E-03)
**CER18:0**	0.26 (1.3E-31)	0.25 (1.7E-29)	0.13 (5.3E-09)	0.12 (9.9E-08)
**CER20:0**	0.18 (2.6E-13)	0.18 (2.9E-13)	0.11 (2.5E-07)	0.11 (1.0E-06)
**CER22:0**	0.23 (1.2E-21)	0.22 (8.7E-22)	0.15 (2.5E-13)	0.16 (2.1E-14)
**CER22:1**	0.2 (1.4E-15)	0.21 (1.2E-17)	0.12 (2.6E-07)	0.12 (6.1E-07)
**CER24:0**	0.09 (4.2E-03)	0.1 (2.1E-03)	0.08 (2.2E-03)	0.09 (5.9E-04)
**CER24:1**	0.08 (4.8E-02)	0.08 (5.1E-02)	0.04 (1)	0.05 (1)
**HCER16:0**	-0.08 (1.9E-02)	-0.08 (1.8E-02)	-0.05 (3.9E-01)	-0.04 (1)
**HCER18:0**	-0.16 (1.8E-10)	-0.14 (1.7E-08)	-0.08 (1.5E-03)	-0.07 (3.8E-02)
**HCER20:0**	-0.14 (2.5E-07)	-0.12 (9.0E-06)	-0.05 (1)	-0.03 (1)
**HCER22:0**	-0.12 (6.4E-06)	-0.11 (2.3E-05)	-0.04 (1)	-0.02 (1)
**HCER24:0**	-0.18 (6.3E-14)	-0.19 (9.6E-15)	-0.08 (3.1E-03)	-0.06 (3.8E-01)
**HCER24:1**	-0.22 (3.0E-21)	-0.21 (8.4E-21)	-0.13 (3.9E-10)	-0.11 (1.6E-06)
**LCER14:0**	-0.31 (9.6E-43)	-0.28 (1.2E-34)	-0.15 (8.4E-13)	-0.15 (1.3E-10)
**LCER16:0**	-0.24 (3.7E-27)	-0.23 (2.2E-24)	-0.15 (5.4E-14)	-0.14 (1.6E-11)
**LCER18:0**	-0.06 (3.5E-01)	-0.07 (2.6E-01)	-0.02 (1.0E+00)	-0.01 (1)
**LCER22:0**	-0.16 (1.1E-10)	-0.15 (1.5E-09)	-0.06 (1.2E-01)	-0.05 (1)
**LCER24:0**	-0.21 (3.9E-19)	-0.2 (1.1E-18)	-0.08 (1.7E-03)	-0.07 (2.7E-02)
**LCER24:1**	-0.27 (9.2E-35)	-0.26 (2.7E-32)	-0.15 (2.5E-13)	-0.14 (8.0E-11)

Model 1: bivariate.
Model 2: adjusted for sample of origin, race, sex, and age.
Model 3: Model 2 plus BMI.
Model 4: Model 3 plus statin use.

Table S2. Standardized regression coefficients of those associations between sphingolipid levels, HOMA-IR, and intake of saturated fat-rich foods

Model A: Linear Regression Model of Sphingolipid levels: Influence of High-Fat Meat, Fast Food, HOMA-IR, and BMI

**SPL Species**	**Fast food**	**Fat meat**	**BMI**	**HOMA-IR**
LCER14:0+16:0+24:1	-0.01 (ns)	-0.03 (ns)	-0.05 (p<0.05)	-0.21(p<0.0001)
LCER/CER22	-0.02 (ns)	-0.04 (ns)	-0.04 (ns)	-0.33 (p<0.0001)
CER18	0.06 (p<0.05)	0.08 (p<0.001)	0.15 (p<0.0001)	0.18 (p<0.0001)
CER22	0.01 (ns)	0.03 (ns)	0.003 (ns)	0.23 (p<0.0001)
LCER14	-0.03 (ns)	-0.01 (ns)	-0.14 (p<0.0001)	-0.20 (p<0.0001)
LCER16	-0.005 (ns)	-0.03 (ns)	-0.04 (ns)	-0.20 (p<0.0001)
LCER24:1	-0.04 (ns)	-0.2 (ns)	-0.11 (p<0.0001)	-0.21 (p<0.0001)

Model B: Linear Regression Model of HOMA-IR: Influence of Sphingolipid levels, High-Fat Meat, Fast Food, and BMI

	**SPL Species**	**Fast food**	**Fat meat**	**BMI**
LCER14:0+16:0+24:1	-0.15 (p<0.0001)	0.05 (p<0.01)	0.07 (p<0.001)	0.50 (p<0.0001)
LCER/CER22	-0.24 (p<0.0001)	0.05 (p<0.05)	0.06 (p<0.01)	0.48 (p<0.0001)
CER18	0.13 (p<0.0001)	0.06 (p<0.01)	0.06 (p<0.01)	0.50 (p<0.0001)
CER22	0.16 (p<0.0001)	0.05 (p<0.01)	0.07 (p<0.001)	0.51 (p<0.0001)
LCER14	-0.15 (p<0.0001)	0.04 (p<0.05)	0.06 (p<0.01)	0.49 (p<0.0001)
LCER16	-0.14 (p<0.0001)	0.05 (p<0.01)	0.08 (p<0.001)	0.50 (p<0.0001)
LCER24:1	-0.15 (p<0.0001)	0.05 (p<0.05)	0.06 (p<0.001)	0.49 (p<0.0001)

All models were adjusted for sex, age, race and sample of origin. The standardized coefficients and their p-values are presented.

Table S3. Estimates of T2D risk based on levels of selected sphingolipid species in PREDIMED.

Predictor	Continuous variable		Q_4 vs Q_1	
	**HR (95% CI)**	**p-value**	**HR (95% CI)**	**p-value**
LCER14:0	0.84 (0.71-1.00)	0.05	0.66 (0.43-1.01)	0.08
LCER16:0	0.85 (0.72-1.01)	0.07	0.69 (0.45-1.08)	0.11
LCER24:1	0.93 (0.78-1.11)	0.44	0.71 (0.46-1.12)	0.14
CER22:0	1.16 (0.98-1.39)	0.09	1.41 (0.90-2.21)	0.12

Adjusted for age, sex, center, intervention group and BMI level.

Table S4. Correlation between selected lipid levels and confounders used in PREDIMED.

Lipid species	Dyslipidemia	Hypertension	Smoking	Glycemia
	Rho (*p-value*)	Rho (*p-value*)	Rho (*p-value*)	Rho (*p-value*)
LCER14:0	0.01 (0.84)	0.02 (0.52)	**-0.09 (0.01)**	**-0.08 (0.013)**
LCER16:0	-0.02 (0.61)	-0.05 (0.17)	0.01 (0.8)	**-0.12 (0.0004)**
LCER24:1	-0.06 (0.072)	**-0.07 (0.038)**	**0.12 (0.0004)**	**-0.1 (0.0021)**
CER22:0	0.01 (0.82)	0.03 (0.32)	0 (0.98)	**0.09 (0.0072)**

Dyslipidemia, hypertension, and smoking were imputed as yes or no, while fasting glucose values were in mg/dL.

Table S5. Estimates of T2D risk based on selected sphingolipid levels in prediabetic patients from PREDIMED.

Time	Predictor as continuous variable	Model I		Model II		Model III	
		HR (95% CI)	p-value	HR (95% CI)	p-value	HR (95% CI)	p-value
T0	LCER14:0 + LCER16:0 + LCER24:1	0.84 (0.70-1.02)	0.073	0.83 (0.68-1.00)	0.050	0.84 (0.66-1.07)	0.167
T0	(LCER14:0 + LCER16:0 + LCER24:1) / CER22:0	0.80 (0.66-0.97)	0.020	0.80 (0.66-0.97)	0.023	0.83 (0.66-1.04)	0.097
T1	LCER14:0 + LCER16:0 + LCER24:1	0.80 (0.63-1.02)	0.072	0.79 (0.62-1.01)	0.061	0.76 (0.58-1.00)	0.051
T1	(LCER14:0 + LCER16:0 + LCER24:1) / CER22:0	0.81 (0.64-1.03)	0.088	0.84 (0.66-1.07)	0.153	0.75 (0.57-0.98)	0.038
**Time**	**Predictor (Q_4 vs Q_1)**	**Model I**		**Model II**		**Model III**	
		**HR (95% CI)**	**p-value**	**HR (95% CI)**	**p-value**	**HR (95% CI)**	**p-value**
T0	LCER14:0 + LCER16:0 + LCER24:1	0.60 (0.34-1.04)	0.068	0.58 (0.33-1.02)	0.058	0.65 (0.32-1.32)	0.233
T0	(LCER14:0 + LCER16:0 + LCER24:1) / CER22:0	0.57 (0.34-0.98)	0.042	0.59 (0.34-1.03)	0.062	0.54 (0.27-1.07)	0.079
T1	LCER14:0 + LCER16:0 + LCER24:1	0.46 (0.23-0.95)	0.036	0.43 (0.21-0.92)	0.030	0.52 (0.23-1.15)	0.107
T1	(LCER14:0 + LCER16:0 + LCER24:1) / CER22:0	0.65 (0.32-1.32)	0.236	0.71 (0.34-1.50)	0.376	0.58 (0.27-1.28)	0.181

Analysis at T0 includes 490 subjects and the analysis at T1 includes 337 subjects, with fasting glucose levels over 100 mg/dL.

- Model I: Age, sex, recruitment center, intervention group and BMI level.
- Model II: Age, sex, recruitment center, intervention group, BMI level, dyslipidemia, hypertension, and smoking.
- Model III: Age, sex, recruitment center, intervention group, BMI level, dyslipidemia, hypertension, smoking and fasting glycemia.
